# The Prevalence and Characteristics of Embolic Stroke of Undetermined Source Among Sudanese Patients From November 2019 to March 2020: A Cross‐Section Study

**DOI:** 10.1002/brb3.70709

**Published:** 2025-07-21

**Authors:** Alaa Abdallah Idris Fadul, Mohammed Khalafalla Saees, Osman Amir

**Affiliations:** ^1^ Department of Neurology, Sudanese National Center for Neurological Sciences Ibrahim Malik Hospital Khartoum Sudan; ^2^ Department of Hematology, Faculty of Medical Laboratory Sciences Karary University Omdurman Sudan

**Keywords:** acute ischemic stroke, embolic stroke of undetermined source, Sudanese

## Abstract

**Aim:**

To study the prevalence and characteristics of embolic stroke of undetermined source (ESUS) among Sudanese patients.

**Methods:**

This cross‐sectional study included 70 patients with recent ischemic strokes at the National Center for Neurological Sciences of Ibrahim Malik Hospital and Omdurman Teaching Hospital in Khartoum from November 2019 to March 2020. Characteristics of ESUS patients were analyzed and compared with stroke of determined source (SDS) patients. Demographics were analyzed, alongside clinical history. Imaging findings from computed tomography (CT) scans, carotid Doppler ultrasound, and echocardiographic evaluations were also assessed.

**Results:**

There were 18 (25.7%) cases in ESUS group and 52 (74.3%) cases in SDS group. ESUS group was significantly younger (44.3 ± 9.9 vs. 62.3 ± 17.7 years; *p* value < 0.001) with female predominance (66.7% vs. 46.2%; *p* value *=* 0.001) than SDS group. Risk factors such as hypertension (44.2%) and diabetes mellitus (DM) (35.6%) were prevalent in the SDS group. In electrocardiography (ECG) findings, left ventricular hypertrophy (LVH) (ESUS = 16.7% vs. SDS = 7.7%) and bradycardia (ESUS = 5.6% vs. SDS = 0%) were common among ESUS patients, while, atrial fibrillation (AF) (ESUS = 0% vs. SDS = 15.4%), ischemic changes (ESUS = 11.1% vs. SDS = 32.7%), and left bundle branch block (LBBB) (ESUS = 0% vs. SDS = 7.7%) were common among SDS patients. Normal echocardiography (64.7% vs. 17.3%; *p* value = 0.003) as well as carotid ultrasonography (US) findings (61.1% vs. 36.5%; *p* value = 0.002) were more prevalent in the ESUS group than in the SDS group. Multivariate analysis identified female gender (OR = 2.33; 95% CI: 1.76–7.16; *p* value = 0.016), normal findings on echocardiography (OR = 8.19; 95% CI: 2.76–24.32; *p* value 0.003), normal carotid Doppler ultrasound (OR = 5.0; 95% CI: 1.87–32.7; *p* value = 0.023), and normal ECG findings (OR = 3.5; 95% CI: 1.17–10.44; *p* value = 0.036) as significant predictors of ESUS.

**Conclusion:**

The study underscores significant differences between ESUS and SDS patients, highlighting the need for tailored clinical approaches based on stroke classification.

## Graphical abstract

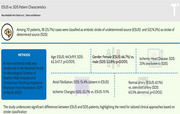

## 1 INTRODUCTION

Embolic stroke of undetermined source (ESUS) has emerged as a significant category within the spectrum of ischemic strokes, representing approximately 9%–25% of all cases. This classification was introduced in 2014 to describe nonlacunar ischemic strokes where no definitive etiology can be identified despite thorough diagnostic evaluations. Unlike traditional stroke categories, ESUS is characterized by its cryptogenic nature, where embolism is presumed to be the primary mechanism but lacks identifiable sources, such as atrial fibrillation (AF) or significant carotid artery stenosis (Hart et al. 2014; Modolo et al. 2019; Ntaios et al. 2024; Sposato et al. 2024).

The clinical implications of ESUS are profound, particularly concerning secondary prevention strategies. Patients with ESUS are typically younger and present with fewer conventional vascular risk factors compared to those with strokes of determined sources. This demographic distinction raises important questions about the underlying pathophysiology and risk profiles associated with ESUS. Studies indicate that these patients experience a relatively high recurrence rate of strokes—approximately 29% within 5 years—which underscores the necessity for effective management strategies (Ntaios et al. 2024; Perkins et al. 2022).

In contrast to strokes of determined source, which often have well‐established risk factors and treatment protocols, ESUS presents unique challenges. Research has shown that the risk factors for ESUS may differ significantly from those associated with other stroke types. For instance, left ventricular dysfunction and aortic arch atherosclerosis have been identified as potential contributors to embolic events in ESUS patients, while traditional risk factors such as hypertension and diabetes are less prevalent in this group (Perkins et al. 2022; Scavasine et al. 2021).

As the understanding of ESUS evolves, it becomes increasingly clear that this condition encompasses a heterogeneous group of patients with diverse underlying mechanisms. This complexity necessitates further research to elucidate specific risk factors and potential therapeutic targets. Considering the characteristics of Sudanese patients with ESUS is critical for developing targeted interventions and improving overall stroke care in the region. This work aims to explore the factors associated with ESUS among Sudanese patients, contributing valuable insights into this understudied area of neurology.

## 2 MATERIAL AND METHODS

### 2.1 Study Design and Subjects

This is a retrospective cross‐sectional hospital‐based study including 70 AIS patients in the National Center for Neurological Sciences of Ibrahim Malik Hospital and Omdurman Teaching Hospital in Khartoum from November 2019 to March 2020.

Eligibility criteria include age 18 years or older and diagnosed with cute ischemic stroke. Exclusion criteria comprise intracranial hemorrhagic diseases or tumors, infections, etc., nonprimary ischemic stroke, other causes of cerebral infarction (such as hypercoagulability, tumors, etc.), and severe organ dysfunction such as liver and kidney dysfunction.

### 2.2 Sample Size and Technique

The sample size was calculated using the formula (*n* = *z* ^2^ × *p* × *q*/*d* ^2^), where *n* represents the sample size, *z* is the standard score (1.96 for 95% confidence), *p* is the estimated prevalence (7%), *q* is 1 minus *p*, and *d* is the margin of error. For this study, a 5% margin of error and a 95% confidence level were applied. Due to the lack of similar studies in Sudan, we referenced research from Oxfordshire, United Kingdom, which reported a 7% prevalence of ESUS (Li et al. 2015). This value was used as the estimated prevalence for our sample size calculation. In addition, 30 patients were excluded from the study as they did not have primary ischemic stroke.

We retrospectively and totally covered all AIS patients fulfil the inclusion criteria admitting to the Department of Neurology during the study period (November 2019 to March 2020) and a total of 70 patients were analyzed in the study. No patients were documented with COVID‐19 during this period.

### 2.3 Data Collection

Patient demographic characteristics including age and sex were collected on admission. Clinical and laboratory information was also collected, including hypertension, diabetes, AF, smoking, total cholesterol, triglyceride, high‐density lipoprotein (HDL), and low‐density lipoprotein (LDL). Diagnosis of hypertension and diabetes mellitus (DM) was defined as having an evident history of disease from interviewing the patient or diagnosis during the current treatment in hospital. AF was defined as having a history of persistent or paroxysmal AF, based on previous electrocardiograms or prolonged heart‐rhythm monitoring during hospitalization. Imaging data, including head computed tomography (CT), ECG, echocardiography, and carotid Doppler ultrasound (US), were interpreted by experienced specialists. The infarct location was recorded.

We used Org 10172 in Acute Stroke Treatment classification criteria proposed by the Cryptogenic Stroke/ESUS International Working Group in diagnosis of ESUS, which is composed of: stroke detected by CT or MRI that is nonlacunar; absence of extracranial or intracranial atherosclerosis, causing ≥ 50% luminal stenosis in arteries supplying the area of ischemia; no major risk cardioembolic source of embolism; and no other specific cause of stroke identified (e.g., arteritis, dissection, migraine/vasospasm, drug misuse) (Hart et al. 2014).

### 2.4 Data Analysis

Data analyzed by Statistical Package for Social Sciences (SPSS; IBM, version 26.0). The results are expressed as the mean and standard deviation (SD) for continuous variables and number and percentages (%) for categorical variables. Kolmogorov–Smirnov normality test was applied to study variables. Chi‐square test was used as a significance test for categorical variables and an independent *t*‐test for continuous variables. Moreover, multivariable regression analysis was applied to detect the predictors of ESUS. All *p* values considered statistically significant at level 0.05 (two‐sided).

### 2.5 Ethical Consideration

This study was conducted in accordance with the principles of the Helsinki Declaration, approved by the Institutional Review Board (IRB) at the Sudan Medical Specialization Board (SMSB) (02/2019). Also, permissions were taken from administration of selected hospitals. Data were collected anonymously by assigning identity numbers in place of names to ensure the protection of patient identities. This information was securely stored in a separate file, and no individual participant references were included in the study reports. Only the study staff had knowledge of the subjects' identities.

## 3 RESULTS

**FIGURE 1:**
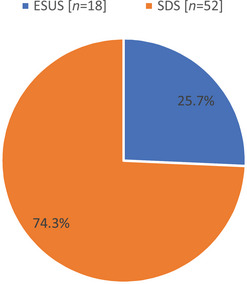
The prevalence of ESUS (*N* = 70). ESUS, embolic stroke of undetermined source; SDS, stroke of determined source.

Among 70 patients, 18 (25.7%) cases were classified as ESUS and 52 (74.3%) cases as stroke of determined source (SDS) (Figure 1)

The average age of the ESUS group was significantly younger than SDS group (44.3 ± 9.9 vs. 62.3 ± 17.7 years; *p* value < 0.001). Gender distribution also differed notably; males were predominant in SDS group (*n* = 28; 53.8%) while females in ESUS (*n* = 12; 66.7%) with statistically significant difference (*p* value = 0.001). The family history of hypertension (*p* value = 0.696), DM (*p* value = 0.436), heart disease (*p* value = 0.636), dyslipidemia (*p* value = 0.363), and stroke (*p* value = 0.399) was not significantly differed between ESUS, SDS, and stroke of atherosclerosis source patients. Lipid profile was not significantly differed between ESUS and SDS patients (*p* value = 0.538). CT imaging findings revealed that right hemispheric infarcts were predominant in both groups, with 66.7% (*n* = 12) in the ESUS cohort and 63.5% (*n* = 33) in the SDS cohort. However, left hemispheric infarcts were more frequently observed in the SDS group (*n* = 17; 32.7%) compared to the ESUS group (*n* = 3; 16.7%). Echocardiographic evaluations showed a significantly higher proportion of normal findings in the ESUS group (*n* = 11; 64.7%) compared to only 17.3% (*n* = 9) in the SDS group (*p* value = 0.003). In addition, ischemic heart disease was identified exclusively within the SDS cohort. Detailed baseline characteristics are shown in Table 1.

**TABLE 1:** The baseline characteristics of the study groups (*N* = 70).

	ESUS (*N* = 18); *n* (%)	SDS (*N* = 52); *n* (%)	*p* value
Age (years); Mean ± SD	44.3 ± 9.9	62.3 ± 17.7	< 0.001^*^ ^a^
Gender			
Male	6 (33.3%)	28 (53.8%)	0.001^*^ ^b^
Female	12 (66.7%)	24 (46.2%)	
Family history of HTN	3 (16.7%)	9 (17.3%)	0.909^b^
Family history of DM	3 (16.7%)	6 (11.5%)	0.290^b^
Family history of heart diseases	0 (0%)	1 (1.9%)	0.560^b^
Family history of dyslipidemia	0 (0%)	1 (1.9%)	0.560^b^
Family history of stroke	0 (0%)	2 (3.8%)	0.398^b^
Lipid profile			
High cholesterol	1 (5.6%)	8 (15.4%)	0.538^b^
High triglyceride	1 (5.6%)	8 (15.4%)	
High LDL	1 (5.6%)	4 (7.7%)	
Low HDL	0 (0%)	4 (7.7%)	
High VLDL	1 (5.6%)	1 (1.9%)	
Computed tomography			
Normal	2 (11.1%)	2 (3.8%)	0.148^b^
Right hemispheric infract	12 (66.7%)	33 (63.5%)	
Left hemispheric infract	3 (16.7%)	17 (32.7%)	
Others	1 (5.6%)	0 (0%)	
Multiplicity			
Single arterial territory	15 (83.3%)	42 (80.8%)	0.712^b^
Multiple arterial territory	3 (16.7%)	10 (19.2%)	
Stroke location			
Anterior circulation	13 (72.2%)	39 (75%)	0.630^b^
Posterior circulation	5 (27.8%)	13 (25%)	
Echocardiography			
Normal	11 (64.7%)	9 (17.3%)	0.003^*^ ^b^
HHD	3 (17.6%)	12 (23.1%)	
IHD	0 (0%)	15 (28.8%)	
Valvular lesions	2 (11.8%)	8 (15.4%)	
Systolic dysfunctions	1 (5.9%)	8 (15.4%)	

Abbreviations: DM, diabetes mellitus; HDL, high‐density lipoprotein; HHD, hypertensive heart disease; HTN, hypertension; IHD, ischemic heart disease; LDL, low‐density lipoprotein; VLDL, very low‐density lipoprotein.

^a^ Independent *t*‐test.

^b^ Chi‐square test.

^*^ *p* value is significant (< 0.05).

**FIGURE 2:**
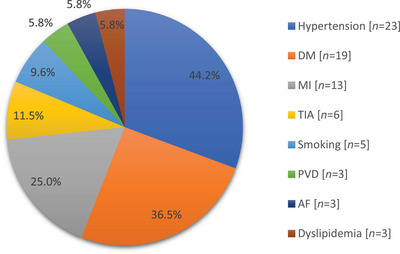
The distribution of risk factors among the patients with SDS (*N* = 52). AF, atrial fibrillation; DM, diabetes mellitus; MI, myocardial infraction; PVD, peripheral vascular disease; TIA, transient ischemic attack.

Among the patients in SDS group, hypertension (*n* = 23; 44.2%), DM (*n* = 19; 35.6%), and old myocardial infraction (*n* = 13; 25%) were the major risk factors as shown in Figure 2.

**FIGURE 3:**
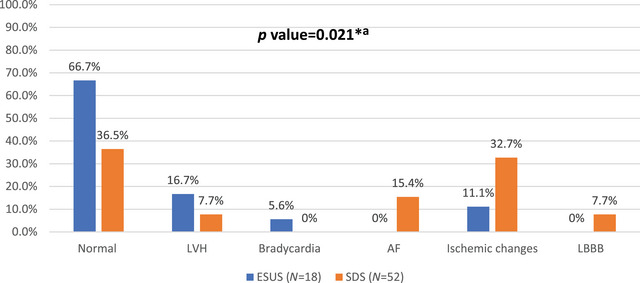
The electrocardiogram findings of the study groups (*N* = 70). AF, atrial fibrillation; ESUS, embolic stroke of undetermined source; LBBB, left bundle branch block; LVH, left ventricular hypertrophy; SDS, stroke of determined source; ^*^ *p* value is significant (< 0.05); ^a^Chi‐square test.

Electrocardiogram results further illustrated differences between the groups; normal ECG findings were present in 66.7% (*n* = 12) of the ESUS patients versus 36.5% (*n* = 19) in the SDS group (*p* value = 0.021). LVH (ESUS = 16.7% vs. SDS = 7.7%) and bradycardia (ESUS = 5.6% vs. SDS = 0%) were common among ESUS patients, while, AF (ESUS = 0% vs. SDS = 15.4%), ischemic changes (ESUS = 11.1% vs. SDS = 32.7%), and LBBB (ESUS = 0% vs. SDS = 7.7%) were common among SDS patients as shown in Figure 3.

**FIGURE 4:**
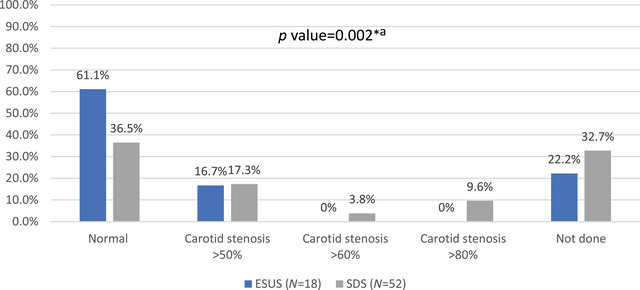
The carotid Doppler ultrasound findings of the study groups (*N* = 70). ESUS, embolic stroke of undetermined source; SDS, stroke of determined source; ^*^ *p* value is significant (< 0.05); ^a^Chi‐square test.

As shown in Figure 4, the Doppler US assessments indicated that normal carotid findings were more prevalent in the ESUS group (*n* = 11; 61.1%) than in the SDS group (*n* = 19; 36.5%), with a significant difference (*p* value = 0.002) indicating a higher incidence of carotid stenosis among those with determined sources.

The multivariate logistic regression analysis identified several significant predictors for ESUS including: female gender (OR = 2.33; 95% CI: 1.76–7.16; *p* value = 0.016), normal findings on echocardiography (OR = 8.19; 95% CI: 2.76–24.32; *p* value = 0.003), normal carotid Doppler US (OR = 5.0; 95% CI: 1.87–32.7; *p* value = 0.023), and normal ECG findings (OR = 3.5; 95% CI: 1.17–10.44; *p* value = 0.036) (Table 2).

**TABLE 2:** Multivariate logistic regression showed the predictor of ESUS.

	OR	95% CI	*p* value
Age	0.92	0.88–0.96	< 0.001
Gender (female)	2.33	1.76–7.16	0.016
Echocardiography (normal)	8.19	2.76–24.32	0.003
Carotid Doppler ultrasound (normal)	5.0	1.87–32.7	0.023
Electrocardiography (normal)	3.50	1.17–10.44	0.036

## 4 DISCUSSION

In this study, we found the prevalence of ESUS was 25.7% (*n* = 18). The prevalence of ESUS is ranged from 17% to 25% (Ntaios et al. 2024). Our rate was comparable to the study of Wang et al. (2020) (27%) and Takasugi et al. (2015) (23.6%), but higher than Ntaios et al. (2015) (10%). This difference might be attributed to the differences in geographical areas, genetic as well biological factors, sampling, and sample sizes.

The average age of the ESUS group was significantly younger at 44.3 years compared to 62.3 years in the SDS group (*p* value < 0.001). This is similar to findings of Scavasine et al. who reported that ESUS patients tend to be younger than those with other stroke types, emphasizing the need for tailored approaches in younger populations who may present with fewer traditional risk factors (Scavasine et al. 2021).

The gender distribution also differed significantly, female gender significantly increased the odds of ESUS, with females having 2.33 times higher odds compared to males (95% CI: 1.76–7.16; *p* value = 0.016). This finding is consistent with existing literature, which often highlights a male predominance in stroke cases associated with established risk factors such as hypertension and ischemic heart disease (Masina et al. 2016). In contrary, with findings from a larger cohort study that indicated a predominance of male patients across both groups but did not specify gender differences as distinctly (Wang et al. 2020).

The study found no significant differences in family history for hypertension, DM, heart disease, dyslipidemia, and stroke among the groups. This agreed with findings from other studies that suggest younger patients with ESUS generally have fewer vascular risk factors compared to older patients (Al Khathaami et al. 2019; Leventis et al. 2020).

Imaging findings revealed that right hemispheric infarcts were predominant in both groups (66.7% in ESUS vs. 63.5% in SDS), while left hemispheric infarcts were more frequently observed in the SDS group (32.7%). This is consistent with literature suggesting that infarct locations can vary based on the underlying etiology; however, previous studies have indicated that bilateral infarcts may be more common in ESUS patients (Lee et al. 2024). The similar rate of normal CT findings suggests that imaging alone may not suffice for definitive diagnosis, a point emphasized by recent consensus statements advocating for comprehensive diagnostic evaluations in suspected ESUS cases.

The echocardiographic evaluations showed a significantly higher proportion of normal findings in the ESUS group (OR = 8.19; 95% CI: 2.76–24.32; *p* value = 0.003). This finding corroborates studies highlighting that echocardiographic abnormalities are more prevalent in patients with determined sources, particularly AF and ischemic heart disease (Ntaios et al. 2024). The presence of ischemic heart disease exclusively within the SDS cohort further emphasizes this distinction.

Normal ECG was associated with 3.50 times higher odds of ESUS (95% CI: 1.17–10.44; *p* value = 0.036). AF was observed solely among patients in the SDS group, which consist with established knowledge that AF is a significant risk factor for cardioembolic strokes (Alshehri 2019). This reinforces the notion that identifying AF is crucial for managing patients with determined sources.

Doppler US assessments indicated that normal carotid findings were more prevalent in the ESUS group highlighting a higher incidence of carotid stenosis among those with determined sources (OR = 5.0; 95% CI: 1.87–32.7; *p* value = 0.023). This finding is consistent with previous studies showing that carotid artery disease is a common etiology for strokes classified as determined sources (Hu et al. 2022).

Several limitations must be acknowledged in this study. First, the relatively small sample size may limit the generalizability of the findings. A larger prospective cohort studies would provide more robust data in both clinical and therapeutic aspects as well as enhance statistical power. In addition, this study's retrospective nature introduces potential biases in patient selection and data collection and due to low source setting of the study we did not performed additional examinations including transthoracic echocardiography (TTE) and transesophageal echocardiography (TEE) to find out other findings like patent foramen ovale (PFO). Moreover, while significant differences were observed, causative relationships cannot be established due to the observational design. The study also did not account for potential confounding variables such as lifestyle factors or genetic predispositions that could influence stroke outcomes.

Despite its limitations, this study offers valuable insights into the differences between ESUS and SDS patients. It highlights critical demographic and clinical characteristics that can inform clinical practice. The use of multiple diagnostic modalities—including imaging, echocardiography, and ECG—provides a comprehensive view of patient profiles and supports a multidimensional approach to stroke management. Furthermore, emphasizing the differences in risk factors and imaging findings contributes to a better understanding of stroke etiology, which is essential for developing targeted secondary prevention strategies. The findings may also guide future research efforts aimed at identifying high‐risk populations within the ESUS category who might benefit from more aggressive treatment protocols.

## 5 CONCLUSION

In conclusion, this study highlights significant differences between patients with embolic strokes of undetermined sources and those with determined sources across various clinical parameters and imaging findings. The younger age, female sex, and lower prevalence of traditional risk factors among ESUS patients suggest distinct underlying mechanisms that may influence management strategies for these two populations. Further research is needed to explore these differences and their implications for clinical practice, particularly regarding preventive strategies tailored to individual patient profiles.

## Author Contributions

**Alaa Abdallah Idris Fadul**: conceptualization, methodology, data curation, investigation, funding acquisition, visualization, project administration, resources, writing–original draft, writing–review and editing. **Mohammed Khalafalla Saees**: conceptualization, methodology, investigation, validation, supervision, writing–review and editing, resources. **Osman Amir**: conceptualization, methodology, software, data curation, formal analysis, supervision, writing–original draft, writing–review and editing.

## Ethics Statement

Ethical approval was obtained from center's ethical committee. Both verbal and written consents to publish this information were gained from the patients.

## Consent

All authors gave their approval for publication.

## Conflicts of Interest

The authors declare no conflicts of interest.

## Peer Review

The peer review history for this article is available at https://publons.com/publon/10.1002/brb3.70709

## Data Availability

The data utilized and analyzed in the current manuscript are available from the authors on reasonable request.

## References

[brb370709-bib-0001] Al Khathaami, A. M. , B. Al Bdah , A. Alnosair , et al. 2019. “Characteristics and Outcomes of Younger Adults With Embolic Stroke of Undetermined Source (ESUS): A Retrospective Study.” Stroke Research and Treatment 2019: 4360787. 10.1155/2019/4360787. 31885851 PMC6914878

[brb370709-bib-0002] Alshehri, A. M. 2019. “Stroke in Atrial Fibrillation: Review of Risk Stratification and Preventive Therapy.” Journal of Family and Community Medicine 26, no. 2: 92–97. 10.4103/jfcm.JFCM_99_18. 31143079 PMC6515763

[brb370709-bib-0003] Hart, R. G. , H. C. Diener , S. B. Coutts , et al. 2014. “Embolic Strokes of Undetermined Source: The Case for a New Clinical Construct.” Lancet Neurology 13: 429–438. 10.1016/S1474-4422(13)70310-7. 24646875

[brb370709-bib-0004] Hu, X. , J. Chen , H. Fu , et al. 2022. “Association Between Carotid Artery Perivascular Fat Density and Embolic Stroke of Undetermined Source.” Frontiers in Neurology 12: 765962. 10.3389/fneur.2021.765962. 35250789 PMC8894862

[brb370709-bib-0005] Lee, I. , J. Heo , H. Lee , et al. 2024. “Long‐Term Outcomes of Patients With Embolic Stroke of Undetermined Source According to Subtype.” Scientific Reports 14: 9295. 10.1038/s41598-024-58292-4. 38653743 PMC11039691

[brb370709-bib-0006] Leventis, I. , K. Perlepe , D. Sagris , et al. 2020. “Characteristics and Outcomes of Embolic Stroke of Undetermined Source According to Stroke Severity.” International Journal of Stroke 15, no. 8: 866–871. 10.1177/1747493020909546. 32122289

[brb370709-bib-0007] Li, L. , G. S. Yiin , O. C. Geraghty , et al. 2015. “Oxford Vascular Study. Incidence, Outcome, Risk Factors, and Long‐Term Prognosis of Cryptogenic Transient Ischaemic Attack and Ischaemic Stroke: A Population‐Based Study.” Lancet Neurology 14: 903–913. 10.1016/S1474-4422(15)00132-5. 26227434 PMC5714616

[brb370709-bib-0008] Masina, M. , A. Cicognani , C. Lofiego , P. R. Malservisi , and A. Lombardi . 2016. “Embolic Stroke of Undetermined Source: A Retrospective Analysis From an Italian Stroke Unit.” Italian Journal of Medicine 10: 202–220. 10.4081/itjm.2016.690.

[brb370709-bib-0009] Modolo, P. , J. De Souza , F. Winckler , et al. 2019. “Embolic Stroke of Undetermined Source (ESUS) Cohort of Brazilian Patients in a University Hospital.” Arquivos De Neuro‐Psiquiatria 77, no. 5: 315–320. 10.1590/0004-282x20190045. 31188994

[brb370709-bib-0010] Ntaios, G. , B. Helmut , D. Wolfram , et al. 2024. “Embolic Strokes of Undetermined Source: A Clinical Consensus Statement of the ESC Council on Stroke, the European Association of Cardiovascular Imaging and the European Heart Rhythm Association of the ESC.” European Heart Journal 45, no. 19: 1701–1715. 10.1093/eurheartj/ehae150. 38685132 PMC11107123

[brb370709-bib-0011] Ntaios, G. , V. Papavasileiou , H. Milionis , et al. 2015. “Embolic Strokes of Undetermined Source in the Athens Stroke Registry: A Descriptive Analysis.” Stroke 46: 176–181. 10.1161/STROKEAHA.114.007240. 25378429

[brb370709-bib-0012] Perkins, J. D. , N. Akhtar , R. Singh , A. Kamran , and S. Ilyas . 2022. “Partitioning Risk Factors for Embolic Stroke of Undetermined Source Using Exploratory Factor Analysis.” International Journal of Stroke 17, no. 4: 407–414. 10.1177/17474930211009847. 33787396 PMC8969073

[brb370709-bib-0013] Scavasine, V. , R. Gustavo , T. Rebeca , et al. 2021. “Embolic Stroke of Undetermined Source (ESUS) and Stroke in Atrial Fibrillation Patients: Not so Different After All?” International Journal of Cardiovascular Sciences 34, no. 5: 517–522. 10.36660/ijcs.20190191.

[brb370709-bib-0014] Sposato, L. A. , N. B. Sur , M. Katan , et al. 2024. “Embolic Stroke of Undetermined Source: New Data and New Controversies on Cardiac Monitoring and Anticoagulation.” Neurology 103, no. 1: e209535. 10.1212/wnl.0000000000209535. 38861698

[brb370709-bib-0015] Takasugi, J. , H. Yamagami , K. Toyoda , and K. Nagatsuka . 2015. “Diagnostic Considerations of Embolic Strokes of Undetermined Source on Admission.” Neurosonology 28: 17–20. 10.2301/neurosonology.28.17.

[brb370709-bib-0016] Wang, W. , X. Tang , W. Liu , K. Jia , X. Zhao , and F. Yu . 2020. “Clinical Features of Embolic Stroke of Undetermined Source.” Frontiers in Neurology 11: 58. 10.3389/fneur.2020.00058. 32117020 PMC7013046

